# Occurrence of Helicobacter Pylori in Specimens of Chronic Gastritis and Gastric Adenocarcinoma Patients: A Retrospective Study at University Teaching Hospital, Kigali, Rwanda

**DOI:** 10.24248/eahrj.v5i2.667

**Published:** 2021-11-15

**Authors:** Theoneste Nizeyimana, Belson Rugwizangoga, Felix Manirakiza, Alvaro C Laga

**Affiliations:** a Department of Clinical Biology, School of Medicine and Pharmacy, College of Medicine and Health Sciences, University of Rwanda, Kigali, Rwanda; b Department of Pathology at University Teaching Hospital of Kigali, Kigali, Rwanda; c Department of Pathology, Brigham and Women's Hospital, Boston, Massachusetts, United States of America

## Abstract

**Introduction:**

*Helicobacter pylori (H. pylori*) infection is the major cause of gastroduodenal diseases in populations of different ages. We conducted aretrospective studyusing archived tissue samples to determine the prevalence of H. pylori infection among patients diagnosed with gastritis and gastric adenocarcinoma by histopathology cases in one hospital in Rwanda.

**Materials and methods:**

Cases of chronic gastritis and gastric adenocarcinoma histologically diagnosed in a tertiary hospital in Rwanda over the period of 2016-2018 were studied for the presence of H. pylori using immunohistochemistry. Diagnosis of positive cases considered immunoreactivity as well as bacterial morphology, including spiral, rod-shaped, angulated and coccoid forms.

**Results:**

Three hundred and seven cases were included in this study; chronic gastritis and gastric adenocarcinoma representing 39% and 61%, respectively. The overall frequency of H. pylori infection was 77.5% (80% among chronic gastritis cases versus 76% among gastric adenocarcinoma cases). Prevalence of *H. pylori* infection in chronic gastritis and adenocarcinoma did not significantly associate with age and sex.

**Conclusion:**

The prevalence of *H. pylori* was high among chronic gastritis and gastric adenocarcinoma cases in Rwanda. Pathologists should investigate the presence of H. pylori in gastric biopsies. Our data shows immunohistochemistry method is feasible and adequate to facilitate detection of H. pylori, which may guide timely treatment.

## BACKGROUND

*Helicobacter pylori (H. pylori*) is a gram-negative bacterium that causes a spectrum of gastroduodenal diseases in humans including chronic gastritis and gastric cancer.^1–3^ Approximately 95% of gastric cancers are adenocarcinomas, which are further histologically categorized into diffuse and intestinal subtypes.^4^ The prevalence of *H. pylori* is approximately 50% of the adult people worldwide.^5–7^ The prevalence is much higher in populations of low socioeconomic status and hygiene level, compared to the developed countries.^8–11^Accordingly, the prevalence of *H. pylori* infection is nearly 30% in the United States of America (USA) adult population, compared to up to 92% in some African regions.^7^^12–14^ This epidemiological trend may explain the over-representation of gastric adenocarcinoma among developing countries (more than 50% of new cases) compared to the developed countries.^4^, ^15–18^

Chronic atrophic gastritis is the earliest pathologic change due to *H. pylori* colonization, and it may eventually lead to gastric cancer.^2^, ^13^, ^19^, ^20^ In *H. pylori-*infected individuals, other factors contributing to chronic atrophic gastritis and cancer include the age at the time of primary infection, as well as the presence of cytotoxin-associated gene A *(cagA)-*positive *H. pylori.*^21^ The prevalence of *H. pylori* infection increases with age, being close to 80% among individuals above 70, whereas it is around 50% in children.^20^,^22^,^23^

Several diagnostic tests are used in the detection of *H. pylori* infection. These include blood serum test, stool antigen test, rapid urease test, urea breath test, detection of *H. pylori* in histopathology specimens, and culture.^5^, ^8^, ^11^, ^12^, ^24^ Histopathology has been shown to have excellent sensitivity and specificity (95% and 99%, respectively), particularly with the use of special and immunohistochemical stains^25^ and it provides additional information about the morphology of the gastric mucosa.^12^,^22–24^ Accordingly, endoscopic biopsiesare used for screening of gastriccarcinoma.^8^, ^16^, ^22^, ^26^

The prevalence of gastroduodenal disease in Rwanda is high, and a recent study using modified rapid urea-se testing during endoscopy showed 75% positivity for *H. pylori.*^27^ Using immunohistochemistry method, this study was performed to determine the frequency of occurrence of *H. pylori* infection in histopathological specimens of patients with chronic gastritis or gastric adenocarcinomain a large teaching hospital of Rwanda.

## MATERIALS AND METHODS

### Study Design and Description

A retrospective descriptive study was conducted in the Anatomical Pathology unit of University Teaching Hospital of Kigali (CHUK). Cases diagnosed as chronic gastritis and gastric adenocarcinoma from 2016 to 2018 were included in this study. Clinical and demographic information and formalin-fixed, paraffin-embedded (FFPE) tissue blocks, and glass slides were retrieved from the archives.

For cases with multiple biopsies from the same patient, the tissue sample with more representative lesion tissue was used. Glass slides were reviewed by 2 independent pathologists to confirm the diagnosis of chronic gastritis or gastric adenocarcinoma. All cases diagnosed as gastric ulcers, gastritis with intestinal metaplasia and/or atrophic gastritis, and chronic gastritis not otherwise specified were included. Sections of tissues with lesion (gastritis or adenocarcinoma) were selected for immunohistochemistry. Patients who met the inclusion criteria, but whose tissue blocks were damaged were excluded.

### Helicobacter pylori detection

Sections (4 μm in thickness) were cut and prepared on charged, frosted glass slides. Immunohistochemistry using a rabbit polyclonal anti-*H. pylori* antibody (DAKO) and the Envision (DAKO) polymer detection system, with diaminobenzidine chromogen and immune-peroxidases according to the manufacturer's specifications was performed. Positive and negative controls were evaluated for each immunostaining assay. Two independent pathologists and one trainee reviewed the immune-stained slides using light microscopes. In case of discrepancy, cases were reviewed and discussed, and the consensus diagnosis agreed by all pathologists. Positivity was ascertained taking into account the presence of immunoreactivity and morphology including spiral, rod-shaped, angulated, and coccoid forms.

## Data management and statistical analysis

Clinical, demographic, and histopathologic diagnosis, including *H. pylori* status, were compiled into a Microsoft Excel sheet. Each patient was assigned an identification code to maintain patients' confidentiality. The data was imported into and analyzed using the Statistical Product and Service Solutions(IBM SPSS). Fisher's exact test was used to compare proportions. A two-tailed *P* value <.05 was considered significant.

## Ethical Considerations

This study was approved by the University of Rwanda (UR), College of Medicine and Health Sciences (CMHS) Institutional Review Board (IRB), approval number 455/CMHS IRB/2019. Permission to access the data was provided by the administration of the hospital.

## RESULTS

### Patients and Disease Characteristics

**FIGURE 1:**
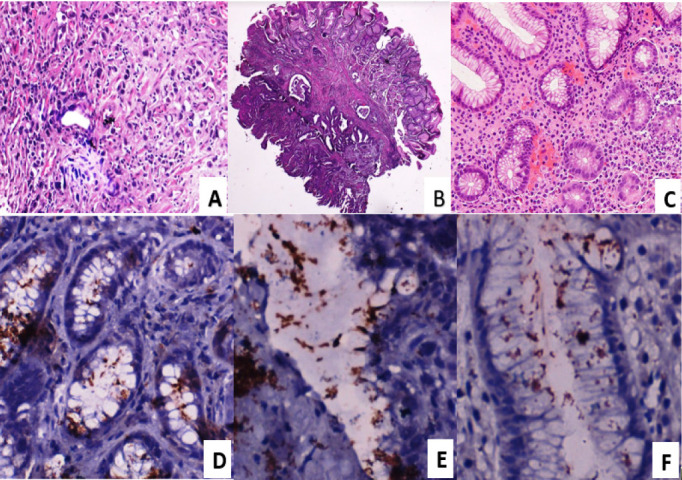
Morphology of gastric adenocarcinoma, chronic gastritis and H. pylori **A:** diffuse type adenocarcinoma (H&E, 200x). **B:** intestinal type adenocarcinoma (H&E, 40x). **C:** chronic gastritis (H&E, 200x). **D:** Coccoid form of H. pylori (H. pylori immunoperoxidase stain, 400x). **E:** Straight rod-shaped form of H. pylori (H. pylori immunoperoxidase stain, 400x). **F:** Spiral and curved form of H. pylori (H. pylori immunoperoxidase stain, 400x).

Most 197(64.2%) patients with chronic gastritis and gastric adenocarcinoma were older than 50 (range=15 to 92, mean=55, median=57) years (Table 1). All the regions of Rwanda are represented, with a slightly higher proportion of patients residing in Kigali City 72(23.5%). Most 264 (86%) biopsies were endoscopic and almost all (98.4%) were taken from the non-cardial (distal) part of the stomach and 61% of cases were diagnosed with gastric adenocarcinoma, with a predominance of intestinal type gastric adenocarcinoma (Table 1). Figure 1 illustrates the routinely stained sections of gastric adenocarcinoma (Figure 1.A-B) and chronic gastritis (Figure 1.C), as well as the various morphologies of *H. pylori* as detected using immunohistochemistry (Figure 1.D-F).

**TABLE 1:** Clinical, Demographic and Pathological Characteristics of 307 Patients with Chronic Gastritis and Gastric Adenocarcinoma

Characteristics	n	%
Age (years, n=307)		
≤50	110	35.8
>50	197	64.2
**Sex (n=307)**		
Male	153	49.8
Female	154	50.2
**Residence (n=307)**		
Kigali city	72	23.5
East	64	20.4
North	61	19.9
South	56	18.2
West	50	16.3
Foreigners	2	0.7
**Anatomical site (n=307)**		
Cardia	5	1.6
Non-cardia	302	98.4
**Specimen type (n=307)**		
Resection specimens	43	14.0
Endoscopic biopsies	264	86.0
**Diagnosis (n=307)**		
Chronic gastritis	121	39.4
**Gastric adenocarcinoma (n=196)** 196	60.6	
Intestinal type	104	55.9
Diffuse type	63	33.9
Mixed type	19	10.2
**H. pylori status (n=307)**		
Positive	238	77.5
Negative	69	22.5

### Biological behavior of chronic gastritis and gastric adenocarcinoma

The prevalence of *H. pylori* infection was 80.2% and 75.8% in chronic gastritis and gastric adenocarcinoma, respectively (Table 2), but there was no significant difference in the number of cases with *H. pylori* infection when comparing chronic gastritis and gastric adenocarcinoma groups of patients (*P*=0.371). Moreover, the type of gastric adenocarcinoma showed no correlation with *H. pylori* infection (*P*=0.732).

**TABLE 2:** Correlation Between H. Pylori and Clinical and Demographic Characteristics Of Patients With Chronic Gastritis and Gastric Adenocarcinoma

Parameter	Characteristics	H. pylori positive		Fisher's exact test P value
		Yes	No	
Diagnosis	Chronic gastritis	97	24	.403
	Gastric adenocarcinoma	141	45	
Age, chronic	<50	42	12	.648
gastritis	≥50	55	12	
Age, gastric	<50	45	9	.136
adenocarcinoma	≥50	96	36	
Sex, chronic	Male	55	14	>.999
gastritis	Female	42	10	
Sex, gastric	Male	65	19	.731
adenocarcinoma	Female	76	26	
Type of	Intestinal	80	24	.732
adenocarcinoma	Non-intestinal	61	21	

The frequency of presence of *H. pylori* in the reviewed biopsies did not varies with the types of studied gastroduodenal diseases (chronic gastritis and adenocarcinoma), age and sex (Table 2).

## DISCUSSION

*H. pylori* plays a major role in gastrointestinal diseases including chronic gastritis, gastroduodenal ulcers, and gastric adenocarcinoma and MALT lymphoma. Previous studies have shown that immunohistochemistry is a sensitive and reliable test for identifying *H. pylori* infection in tissue sections.^13^, ^25^ In the present study, we analyzed the proportion of *H. pylori* infection among pathology samples of patients diagnosed with chronic gastritis and gastric adenocarcinomain one of the hospitals in Rwanda.

The high proportion of endoscopic biopsies (86%) in our cohort is in keeping with the fact that endoscopic biopsy is considered a gold standard procedure for the screening and the detection of gastric cancer.^1^, ^20^, In both patient groups, the majority of cases were older than 50 years of age, while both sexes were almost equally represented. The relatively over-representation of Kigali and the Eastern regions among the cohort may be explained by the geographical accessibility to the study site.

The overall prevalence of *H. pylori* infection among both disease groups was high (77%). Asimilarproportion (75%) of *H. pylori* infection (using modified rapid urease test) was previously reported in Rwanda, in a study comprising all patients who underwent upper gastrointestinal endoscopy. The proportions, although slightly different, are not significantly different.^27^ The proportion of *H. pylori* infection among various cohorts of individuals in Africa ranges from 55 to 92%.^11^, ^14^ In addition, there was no significant difference in the rates of *H. pylori* infection between chronic gastritis and gastric adenocarcinoma (*P*= 0.371). These findings are similar to those previously reported in other settings.^14^

In the present study, although there was no significant association between age (using a 50-year cut-off) and *H. pylori* infection, a trend towards a higher proportion of *H. pylori* infection with increasing age among chronic gastritis patients was observed. In contrast, it tends to de-crease with increasing age among gastric adenocarcinoma patients. These findings are consistent with the previous studies which reported that *H. pylori* infection is typically universal in all adulthood age groups^20^, ^28^, because it is up taken during youthfulness and generally persists during lifetime except if correctly managed.^29^

## CONCLUSION

This study documents a high prevalence of *H. pylori* infection in pathology specimens at one major hospital in Rwanda. Study findings indicate that all age and both sexes are at risk of getting H. pylori infection, and suggest that pathologists should consider using immunohistochemistry in the evaluation of gastric biopsies. This may allow early detection and appropriate treatment, and hence decrease the risk of gastric cancer.
